# Development of 3D Printable Calcium Phosphate Cement Scaffolds with Cockle Shell Powders

**DOI:** 10.3390/ma16186154

**Published:** 2023-09-10

**Authors:** Eunbee Cho, Jae Eun Kim, Juo Lee, Sangbae Park, Sungmin Lee, Jong Hoon Chung, Jungsil Kim, Hoon Seonwoo

**Affiliations:** 1 Department of Agricultural Machinery Engineering, College of Life Sciences and Natural Resources, Sunchon National University, Suncheon 57922, Republic of Korea; abc2865@hanmail.net; 2 Korea Testing & Research Institute, Suncheon 58023, Republic of Korea; 3 CHA Advanced Research Institute, CHA University, Seongnam 13488, Republic of Korea; je6740@snu.ac.kr; 4 Department of Animal Science & Technology, College of Life Sciences and Natural Resources, Sunchon National University, Suncheon 57922, Republic of Korea; juolee23@naver.com; 5 Interdisciplinary Program in IT-Bio Convergence System, Sunchon National University, Suncheon 57922, Republic of Korea; 6 Department of Convergence Biosystems Engineering, College of Agriculture and Life Sciences, Chonnam National University, Gwangju 61186, Republic of Korea; sb92park@snu.ac.kr; 7 Department of Rural and Biosystems Engineering, College of Agriculture and Life Sciences, Chonnam National University, Gwangju 61186, Republic of Korea; 8 Interdisciplinary Program in IT-Bio Convergence System, Chonnam National University, Gwangju 61186, Republic of Korea; 9 Department of Mechanical Engineering, College of Engineering, Sunchon National University, Suncheon 57922, Republic of Korea; leecm1009@naver.com; 10 ELBIO Inc., Seoul 08812, Republic of Korea; jchung@snu.ac.kr; 11 Department of Biosystems Engineering, College of Agriculture and Life Sciences, Seoul National University, Seoul 08826, Republic of Korea; 12 Department of Bio-Industrial Machinery Engineering, College of Agriculture and Life Sciences, Kyungpook National University, Daegu 41566, Republic of Korea; 13 Department of Convergent Biosystems Engineering, College of Life Sciences and Natural Resources, Sunchon National University, Suncheon 57922, Republic of Korea

**Keywords:** 3D printing, cockle shell, calcium phosphate cement scaffold, bone substitute

## Abstract

Three-dimensional (3D) printed calcium phosphate cement (CPC) scaffolds are increasingly being used for bone tissue repair. Traditional materials used for CPC scaffolds, such as bovine and porcine bone, generally contain low amounts of calcium phosphate compounds, resulting in reduced production rates of CPC scaffolds. On the other hand, cockle shells contain more than 99% CaCO_3_ in the form of amorphous aragonite with excellent biocompatibility, which is expected to increase the CPC production rate. In this study, 3D-printed cockle shell powder-based CPC (CSP-CPC) scaffolds were developed by the material extrusion method. Lactic acid and hyaluronic acid were used to promote the printability. The characterization of CSP-CPC scaffolds was performed using Fourier transform infrared spectra, X-ray diffraction patterns, and scanning electron microscopy. The biocompatibility of CSP-CPC scaffolds was evaluated using cell viability, Live/Dead, and alkaline phosphatase assays. In addition, CSP-CPC scaffolds were implanted into the mouse calvarial defect model to confirm bone regeneration. This study provides an opportunity to create high value added in fishing villages by recycling natural products from marine waste.

## 1. Introduction

Tissue engineering is a process that begins with cell migration and recruitment, followed by cell proliferation, differentiation, and matrix formation [1]. Tissue engineering scaffolds for bone replacement contain biocompatible structures with interconnecting pores that induce cell adhesion and provide an environment conducive to bone tissue formation. Interconnecting pores are important components of tissue engineering scaffolds because they play a role in cell survival, migration, proliferation, and differentiation [2,3]. By precisely controlling the pore size and structure, 3D-printed bone tissue scaffolds can provide a stable microenvironment for cell proliferation. At the same time, tissue-engineered scaffolds with pore sizes > 300 μm can provide a good growth environment for cells and promote nutrient exchange [4]. Common methods of preparing such scaffolds include electrospinning [5,6], lyophilization [7,8], and similar methods. However, these methods have limited reproducibility and versatility in the fabrication process [9,10].

To overcome this problem, three-dimensional (3D) printing technology has been used to fabricate scaffolds by stacking materials. Such 3D printing technology controls the structure of the fabricated scaffold, which consists only of interconnected networks, as well as the shape of the scaffold [11]. The 3D printing method has also been widely used for calcium phosphate cement (CPC) scaffolds, a type of bone substitute [12,13]. CPC scaffolds have been extensively studied due to their excellent biocompatibility, bioactivity, and bone conduction properties [14]. CPC scaffolds offer not only the potential to mimic bone mineralogy, but also stability when molded into bone defects and cured in situ. Therefore, CPC scaffolds may be an excellent candidate for use in bone defects [13]. In particular, 3D-printed CPC scaffolds can provide patient-specific advantages for cranial reconstruction [15]. Their mechanical strength can be increased by adding collagen, and their printability can be improved by adding polymers such as polylactic acid (PLA) [16].

As a component for CPC scaffolds, β-tricalcium phosphate (β-TCP), a form of calcium phosphate, is known to form new bone predominantly with osteoconductivity. In addition, calcium carbonate (CaCO_3_) has excellent properties, including biocompatibility, bioactivity, and high bone conductivity for bone grafting and regeneration [17]. The CaCO_3_ is commonly used in CPC scaffolds in the form of synthetic raw materials [18]. In contrast to synthetic materials, components derived from natural products such as eggshells, animal bones, natural corals, and seashells can reduce the production cost and have positive effects on cytotoxicity, adhesion, and differentiation compared to synthetic materials [19]. Among them, cockle shell is a rich source of CaCO_3_ (95% to 99%) in the form of amorphous aragonite, which has excellent bioavailability [20]. Several studies have reported that denser aragonite is more suitable for biomaterials because it can be more easily incorporated and resorbed by bone tissue [21,22,23]. For this reason, the CPC composites formed by cockle shell and β-TCP exhibited favorable properties for cell proliferation and osteogenic differentiation and bone tissue regeneration due to their bone-like components [14,24]. Therefore, cockle shell may be a promising material for use in 3D-printed CPC scaffolds.

However, CSP must overcome several obstacles to be used in 3D-printed CPC scaffolds. One major issue is low injectability. Since the materials are printed through the nozzle, injectability must be ensured to increase printability [25,26]. There are several approaches to address this issue: (1) changing the particle size, distribution, and shape by powder grinding; (2) adding additives for particle–particle interaction; (3) increasing the viscosity of the binder; and (4) adjusting the extrusion parameters, which can increase the extrusion speed and decrease the residence time to prevent pre-extrusion hardening [27]. Among them, the addition of a biocompatible polymer such as polyethylene glycol (PEG) to the blend can easily overcome the sticking problem, because the addition of additives is easier than other methods [28,29].

In this study, 3D-printed cockle shell powder-incorporated CPC (CSP-CPC) scaffolds were developed using the material extrusion method with sophisticated nozzles. The rheological properties of CSP-CPC scaffolds with different CaCO_3_/cockle shell powder (CC/CS) ratios were analyzed. Then, the printability, degradation, and mechanical properties of CSP-CPC scaffolds were investigated. The surfaces of CSP-CPC scaffolds, which are important for cell adhesion and migration, were observed by scanning electron microscopy (SEM). Then, in vitro studies including cell viability, motility, proliferation, and osteogenic differentiation capacity were conducted. An in vivo study was also performed to confirm bone formation in animal models. A mouse calvarial defect model was used to evaluate biocompatibility in vivo, and micro-CT imaging was used to evaluate bone regeneration [30].

## 2. Materials and Methods

### 2.1. Materials

Calcium phosphate tribasic (β-TCP, Ca_3_(PO_4_)_2_; 2019-1405), polyethylene glycol 6000 (PEG, H(OCH_2_CH_2_)_n_OH; 6554-1400), lactic acid (C_3_H_6_O_3_; 5057-4405), and calcium carbonate (CaCO_3_; 2505-4405) were purchased from DAEJUNG CHEMICALS&METALS (Siheung, Republic of Korea). Hydroxyapatite (Ca_10_(PO_4_)_6_(OH)_2_; 21223), calcium hydrogen phosphate dihydrate (DCPA, CaHPO_4_·2H_2_O; 307653), and sodium phosphate dibasic (Na_2_HPO_4_; S5136) were purchased from Sigma-Aldrich (St. Louis, MO, USA). Hyaluronic acid ((C_14_H_21_NO_11_)n; 8806383168540) was purchased from Saero Hands (Seoul, Republic of Korea). Cockle shells were collected from the southern coast of the Republic of Korea.

### 2.2. Fabrication of 3D-Printed CSP-CPC Scaffolds

The solutions of Na_2_HPO_4_, PEG, hyaluronic acid, and lactic acid were prepared by adding each powder to distilled water. The resulting aqueous solution was homogenized for 6 h. CPC scaffolds were prepared using a mixture of β-TCP, hydroxyapatite, DCPA, CaCO_3_, cockle shell, and aqueous solution (Table 1). The ratio (L/P) of CPC powder to aqueous solution was set at 0.8 mL/1 g. A hand-built 3D printing system with a 25 G (250 μm) tapered needle was used to facilitate the micro-extrusion printing method. After printing, the samples were incubated in a humidified CO_2_ incubator at 37 °C for 24 h.

### 2.3. Characterization

#### 2.3.1. Anti-Washout Test

An anti-washout test was utilized to assess the water resistance of CSP-CPC scaffolds. Each prepared CPC was prepared and placed into a 35 mm polystyrene dish with 5 mL of sterile phosphate-buffered saline (PBS; Welgene Inc., Gyeongsan, Republic of Korea) and stored in a humidified CO_2_ incubator at 37 °C for 24 h. Afterward, the samples were visually assessed.

#### 2.3.2. Printability

The printability of the prepared CSP-CPC scaffolds was assessed using a fluorescence microscope (Nikon, Tokyo, Japan). The same printing conditions of CSP-CPC scaffolds were applied for all mixing ratios. The optimal pressure of each ratio was determined to achieve the uniform extrusion of strands.

#### 2.3.3. Fourier-Transform Infrared Spectroscopy

Fourier-transform infrared spectroscopy (FT-IR) spectra of CSP-CPC scaffolds were measured in the range of 4000 to 400 cm^−1^ using Spectrum Two (PERKIN ELMER, Waltham, MA, USA).

#### 2.3.4. X-ray Diffraction

X-ray diffraction (XRD) was conducted to identify the crystalline and amorphous regions of the scaffolds. An X-ray diffractometer (XRD-7000; SHIMADZU, Kyoto, Japan) was used, operating at 3 kW and with a 2θ range of 10–145° at 10° min^−1^.

#### 2.3.5. Scanning Electron Microscopy

A field emission electron microscope (FE-SEM; JSM-7100F; JEOL, Akishima, Japan) was used to observe the surface morphology of CaCO_3_, CSPs, and CSP-CPC scaffolds. Prior to SEM measurements, all samples were Au-coated at 15 mA using a sputter coating device.

#### 2.3.6. Degradation Test

The CSP-CPC scaffolds underwent a degradation test, which involved incubating them in simulated body fluids (SBFs) at 37 °C for a maximum of three weeks. The SBFs were replaced every three days. The samples that were cultured for either 14 or 21 days were freeze-dried utilizing a freeze dryer. To calculate the degradation value, W_d_ (%) was applied, as shown in Equation (1), where W_before_ and W_after_ were the weights of samples before and after degradation, correspondingly.

W_d_ (%) = (W_before_ − W_after_) × 100 (1)

### 2.4. In Vitro Study

#### 2.4.1. Cell Culture and Seeding

Human dental pulp stem cells (DPSCs) were obtained from a patient’s tooth at the Dental Hospital of Seoul National University (IRB: CRI05004). The DPSCs were cultured on CPC scaffolds with varying concentrations of CSP (*w*/*v*). The cells were then incubated with alpha-modified Eagle’s medium (α-MEM; Welgene Inc., Gyeongsan, Republic of Korea) supplemented with 10% fetal bovine serum (FBS; Welgene Inc., Gyeongsan, Republic of Korea), 2 mM L-glutamine, 100 U/mL penicillin, and 100 μg/mL streptomycin (Gibco BRL, Carlsbad, CA, USA), at 37 °C in a humidified atmosphere with 5% CO_2_. For the osteogenic differentiation of DPSCs, cells were replaced with osteogenic differentiation-conditioned medium composed of α-MEM supplemented with 10% FBS, 1% penicillin, 0.1 μM dexamethasone (Sigma-Aldrich, St. Louis, MO, USA), 10 mM β-glycerophosphate (Sigma-Aldrich, St. Louis, MO, USA), and 100 μM ascorbic acid (Sigma-Aldrich, St. Louis, MO, USA). The culture medium was changed every 2 days.

#### 2.4.2. Cell Viability

The DPSCs were grown in 96-well plates filled with CSP-CPC scaffolds at a density of 2 × 10^4^ cells/well. Cytotoxicity was evaluated using a water-soluble tetrazolium salt assay kit (WST-1; Dogen-bio, Seoul, Republic of Korea). After 1, 3, and 7 days of incubation, the cells were washed with PBS and incubated for 1 h in a medium with 10% WST-1 reagent. Absorbance was measured at 450 nm using a microplate reader.

To evaluate the adhesion and viability of DPSCs seeded on CSP-CPC scaffolds, the Live/Dead Viability/Cytotoxicity Assay Kit (Invitrogen, Waltham, MA, USA) was used. Once the medium was removed and samples were washed once with PBS, a new medium with dye solution containing 2 mM ethidium homodimer^−1^ and 4 mM calcein-AM was added to each well, followed by a 30-min incubation period. The live and dead cells on the scaffolds were observed using a fluorescence microscope (Nikon, Tokyo, Japan).

#### 2.4.3. Osteogenic Differentiation

The osteogenic potential of DPSCs was evaluated with an alkaline phosphatase (ALP) assay kit (ab83369, Abcam, UK). The ALP activity was measured 3 and 5 days after introducing the cells to osteogenic differentiation medium, per the manufacturer’s protocol. To verify the expression of osteogenic markers, DPSCs were seeded onto the CSP-CPC scaffolds in 96-well plates at a density of 2 × 10^4^ cells/well and incubated in humidified CO_2_ for 24 h. After 7 and 14 days of culture, the osteogenic differentiation medium was removed and cells on the CSP-CPC scaffolds were fixed with 4% paraformaldehyde solution (158127, Sigma-Aldrich, St. Louis, MO, USA) for 30 min at room temperature. Samples were treated with 0.2% Triton X-100 (X-100, Sigma-Aldrich, St. Louis, MO, USA) for 15 min, followed by staining with TRITC-conjugated phalloidin (Millipore, Burlington, MA, USA) for 1 h. Monoclonal anti-osteopontin (OPN) antibody (1:500, ab8448, Abcam, Cambridge, UK) and FITC-conjugated goat anti-human antibody were used to stain the OPN protein for 1 h. The cells were then stained with 4′,6-diamidino-2-phenylindole (DAPI; Millipore, Burlington, MA, USA) for 10 min. Images of stained cells were captured using a fluorescence microscope.

### 2.5. In Vivo Study

The bone regeneration ability of CSP-CPC scaffolds was assessed through a mouse calvarial defect model. The animal study protocol was approved by the Institutional Animal Care and Use Committee of Sunchon National University (No. SCNU IACUC 2020-15). Institute of Cancer Research (ICR) mice (4 weeks old) were purchased from Orient Bio (Republic of Korea). A total of 32 mice (6 weeks old) were divided into four groups according to the implantation materials: control (no scaffold), CC10, CC5CS5, and CS10 scaffolds. Anesthesia in mice was induced through the intraperitoneal injection of a fresh mixture of 60 mg/kg of alfaxalone (Alfaxan, Jurox, Australia) and 10 mg/kg of xylazine (Rompun, Republic of Korea). Subsequently, the hair in the bregma region of the skull was removed using a depilatory agent. The exposed scalp was incised approximately 1.5 cm along the midline. A defect was created on the left parietal bone using a dental handpiece and a 2.7 mm diameter trephine bur. The incision site was washed with PBS, and 3D-printed CSP-CPC scaffolds were affixed to the defect site after removal of the trephined calvarial disks. Finally, the incision was sutured. Then, the mice were sacrificed for micro-CT imaging 6 weeks after surgery. The skulls were isolated and scanned with a Skyscan 1272 scanner (Bruker- micro-CT, Konich, Belgium). Subsequently, standardized data were reconstructed using the NRecon software (Bruker-micro-CT) and bone regeneration ability was quantitatively analyzed by the CTAn software (Bruker-micro-CT).

## 3. Results and Discussion

### 3.1. Characterization of CSP-CPC Scaffolds

**Figure 1:**
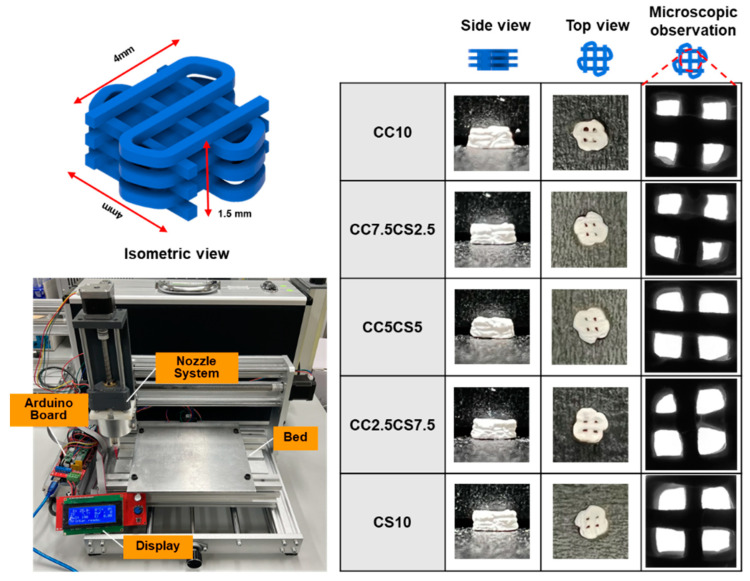
The 3D printer and 3D modeling of scaffold fabrication and various views of 3D-printed scaffolds.

The conditions of 3D printing relied on the thickness and shape of the structures to be extruded. Figure 1 displays the optical observations of the 3D-printed CSP-CPC scaffolds. Constructs with a height of 1.5 mm and a diameter of 4 mm were 3D-printed via a nozzle that had a diameter of 250 μm. The printing speed was optimized to achieve minimal defects in the struts of each layer, at 60 mm/s. The composites prepared under optimized pressure were observed through a microscope to confirm that the strand’s thickness was approximately ~260 μm.

**Figure 2:**
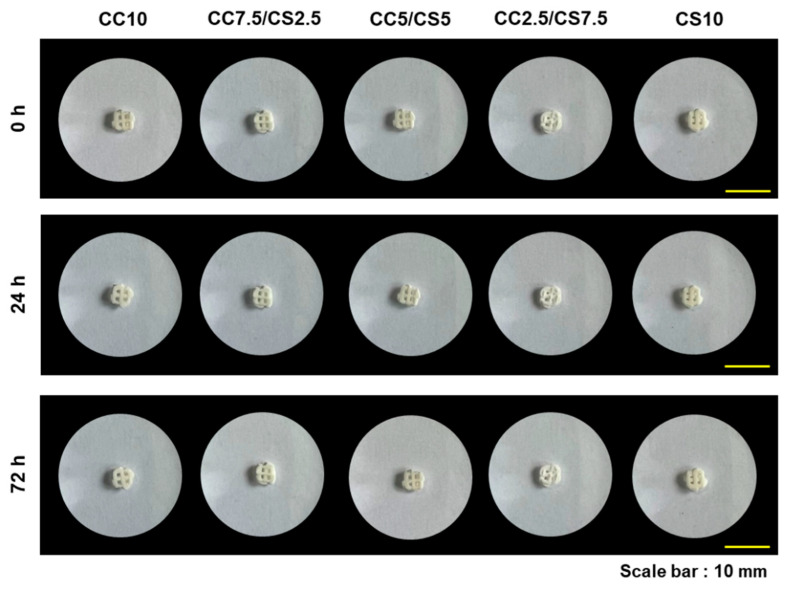
Anti-washout image of CSP-CPC scaffolds at 0, 24, and 72 h of incubation in PBS.

The cohesion of the CSP-CPC scaffolds was assessed by examining the washout resistance. The CSP-CPC scaffolds preserved their shape in all conditions after being immersed in PBS and inspected at 0, 24, and 72 h. (Figure 2). An effective CPC scaffold ought to possess enough fluidity to be injected through needles and strong anti-washout properties to resist disintegration during implantation. The hyaluronic acid, PEG, and lactic acid were capable of maintaining the cements paste’s low viscosity and subsequently elevating it as soon as it was printed. In addition, lactic acid and hyaluronic acid are often used as binding agents or plasticizers in CPC scaffolds. CPC scaffolds are frequently employed in the medical field due to their inherent biodegradability. Lactic acid, being a naturally occurring organic acid in the body, not only promotes the dissolution and recrystallization of calcium phosphate complexes but also enhances its biodegradable properties.

**Figure 3:**
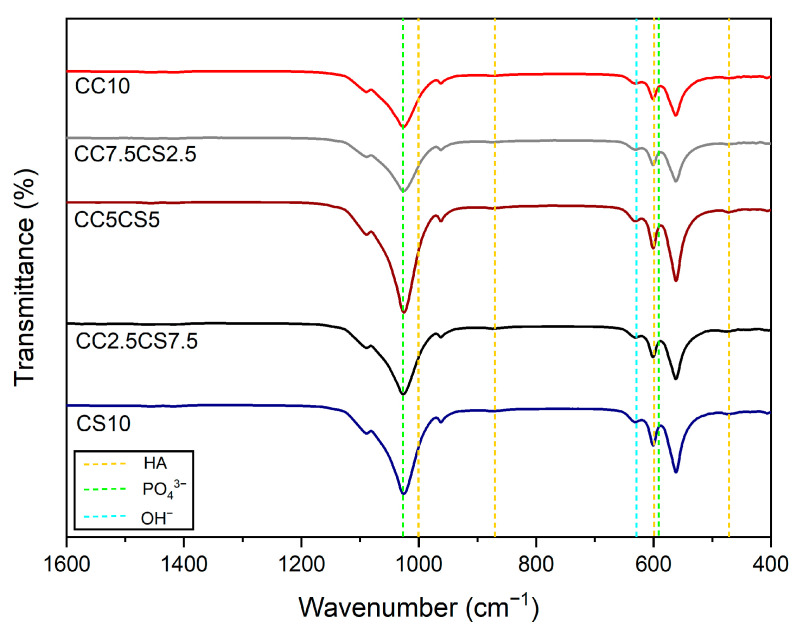
FT−IR spectra of CSP-CPC scaffolds.

Figure 3 shows the FT-IR spectrum for the chemical analysis of the functional groups of specific bonds in the sample. The FT-IR spectra of the CSP-CPC scaffolds confirm that all samples are present in the same stretch. The characteristic peak indicates the stretching mode of the hydroxyl group (625 cm^−1^), and the phosphate groups (1112, 1030, 960, 605, and 563 cm^−1^) indicate the identification of a typical peak characteristic of the CPC scaffolds. This FT-IR analysis confirms the stable interaction of the scaffold components. To confirm the binding properties of hyaluronic acid and lactic acid, further studies are needed to observe the peak or size alterations of CSP-CPC scaffolds when hyaluronic acid and lactic acid are not present individually.

**Figure 4:**
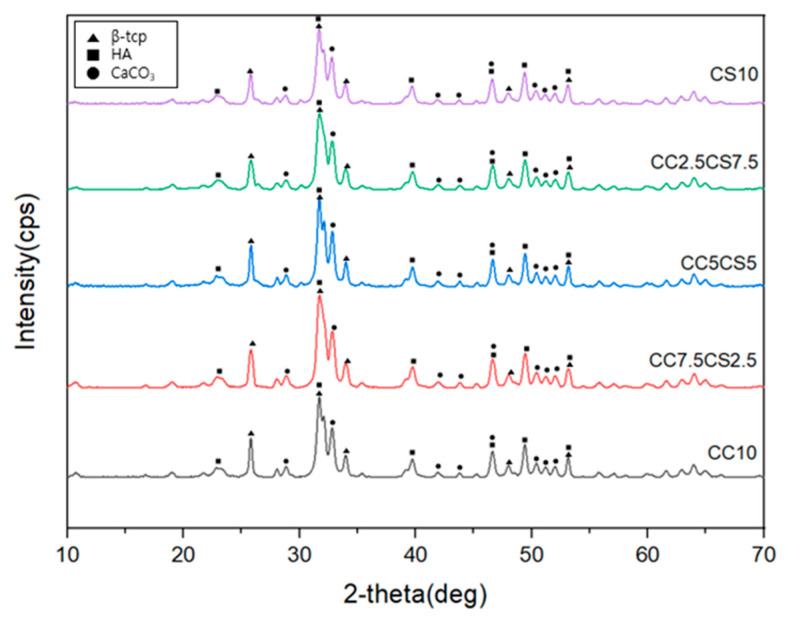
XRD of CSP-CPC scaffolds.

The XRD pattern for the CSP-CPC scaffolds is presented in Figure 4, showing peaks of β-TCP, HA, and CaCO_3_ in all situations. The appearance of β-TCP was confirmed by a prominent diffraction peak between 25 and 33°, while the principal diffraction peak at 31.4° corresponded to HA. Remarkably, along with the diffraction peaks of HA and β-TCP, the slight CaCO_3_ peaks were broadened and overlapping. According to the XRD pattern results, it is not clear which component is dominant, whether it is β-TCP, CaCO_3_, or HA. Additionally, no significant trends were identified regarding an increase or decrease in β-TCP content. The lack of differentiation arises from the fact that the compositions of each group are approximately 90% similar, with the remaining materials consisting of carbonate-based substances such as CaCO_3_ and naturally occurring carbonate-based products such as cockle shells. This suggests that calcium carbonate from cockle shells can be a viable component of CPC scaffolds.

**Figure 5:**
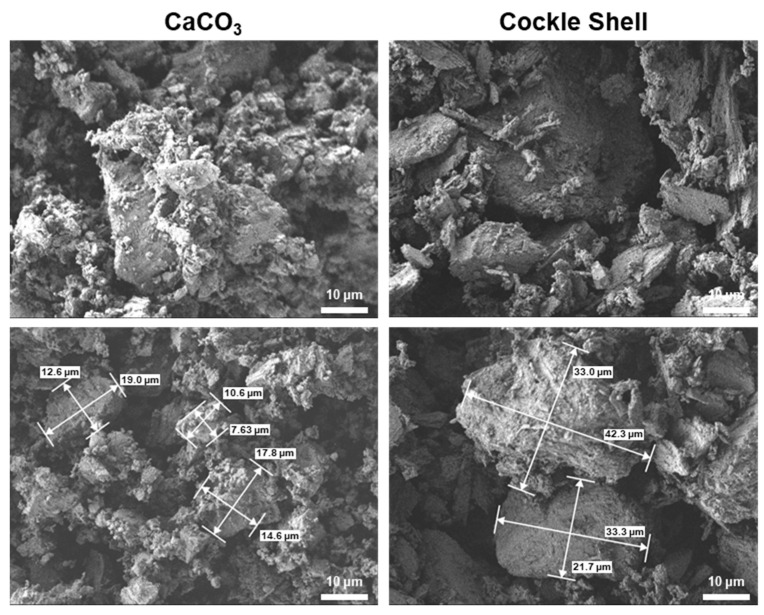
SEM images of CaCO_3_ and cockle shell powders.

**Figure 6:**
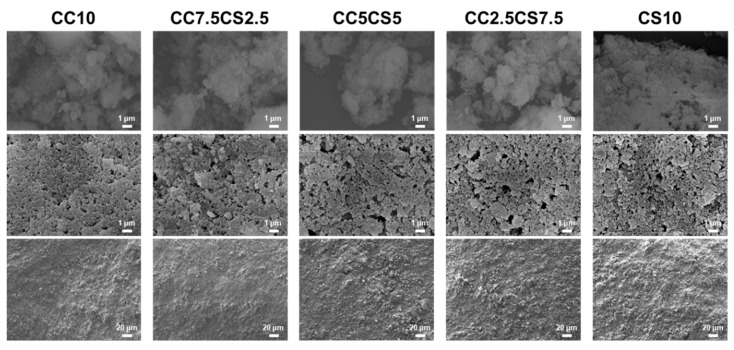
SEM images of surface morphology of CSP-CPC scaffolds.

Through SEM images, it was determined that the particle size of CaCO_3_ powder and that of the CSP had reached a comparable level (Figure 5). As a result, the extrusion of the CSP-CPC scaffolds successfully completed without experiencing any nozzle clogging issues. The SEM image presents the surface morphology of the CSP-CPC scaffolds, which displays a uniformly porous structure where crystals from different materials interlace, forming a cohesive cement microstructure (Figure 6). As the cockle shell proportion increased, the particle size also increased. Additionally, all groups had a Ca/P ratio that exceeded that of hydroxyapatite (Ca/P = 1.67), which is appropriate for bone replacement (Table 2). Further research is needed to analyze the structural homogeneity using scatter plots obtained from SEM images and considering factors such as the pore diameter or wall thickness.

**Figure 7:**
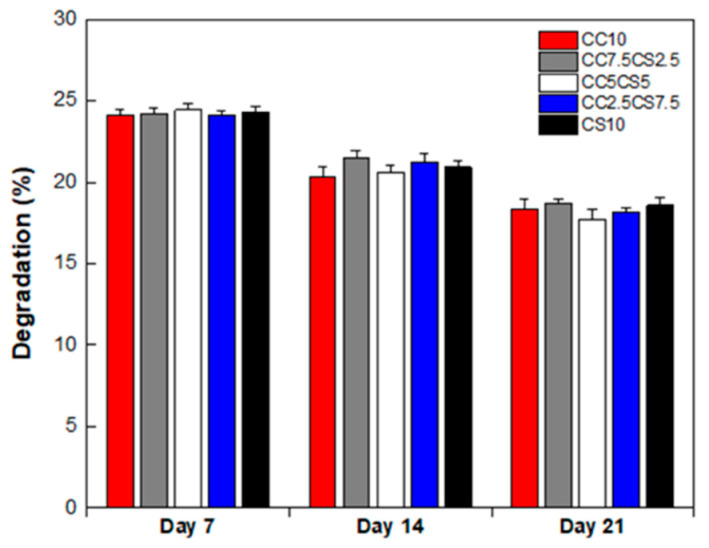
Degradation CSP-CPC scaffolds in SBFs at 37 °C.

The degradation properties of the CSP-CPC scaffolds were assessed via simulated body fluid tests. Due to the significant use of CPC scaffolds in the medical domain, tests with simulated body fluids are typically performed. These fluids are carefully formulated to mimic the interactions present in biological systems, enabling the dissolution and degradation of CPC scaffolds to occur over time. This simulation offers insights into its behavior upon contact with bodily fluids. During tissue regeneration, it is essential for implant materials to be degraded in damaged tissue and subsequently replaced by new tissue. To measure the degradation of samples cultured in SBF solution over a period of 3 weeks, lyophilized samples were weighed to obtain W_d_ (Figure 7). The weight loss in the CSP-CPC scaffolds under all conditions was altered, exhibiting an overall decrease without a significant difference.

### 3.2. In Vitro Test Results

**Figure 8:**
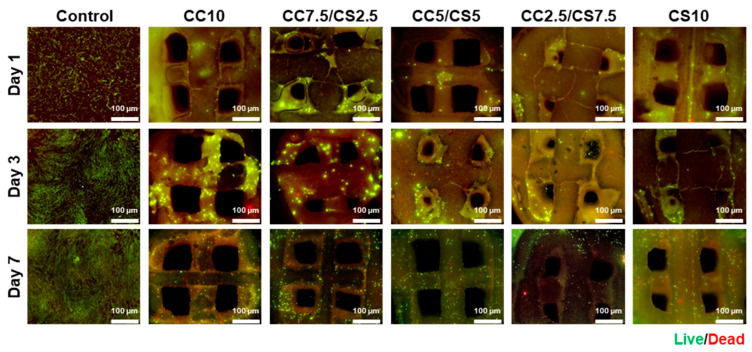
Live/Dead staining images of DPSCs seeded on the CSP-CPC scaffolds.

Figure 8 shows the adhesion and viability of DPSC cells seeded on CSP-CPC scaffolds, measured via the Live/Dead assay. Analysis after 1, 3, and 7 days of incubation showed that cells were distributed throughout the porous structures, with adhesion and diffusion observed. In addition, cell migration occurred within the porous matrix.

**Figure 9:**
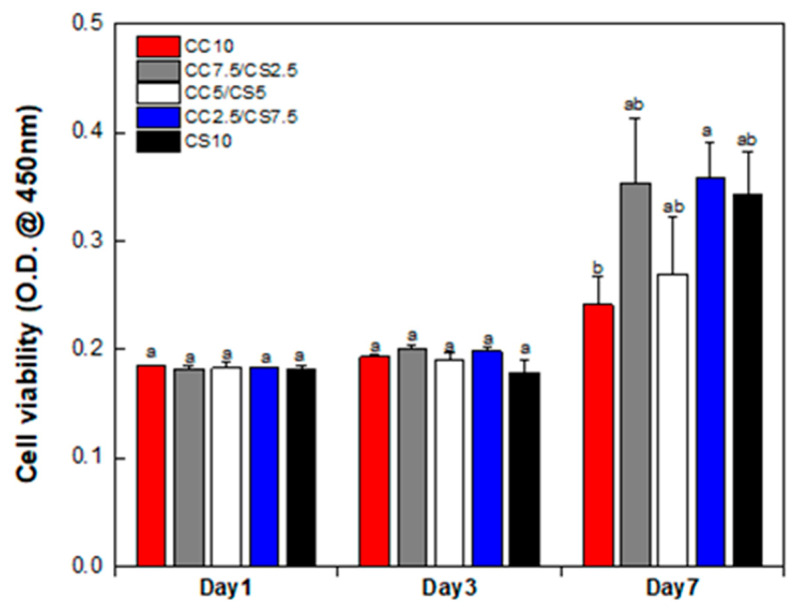
Cell viability of DPSCs on day 1, 3, and 7 of culture using a WST-1 assay (n-5, ANOVA, Duncan’s multiple range test, *p* < 0.05). Error bars indicate the standard error, and different letters indicate that the samples are statistically different.

Cell proliferation on the scaffold was analyzed through measurement of the absorbance of the reaction solution by means of the WST-1 assay (Figure 9). Absorbance was measured after 1, 3, and 7 days. The proliferation rate gradually increased in the CC7.5CS2.5, CC2.5CS7.5, and CS10 groups, with the CC2.5CS7.5 group exhibiting the highest proliferation rate.

**Figure 10:**
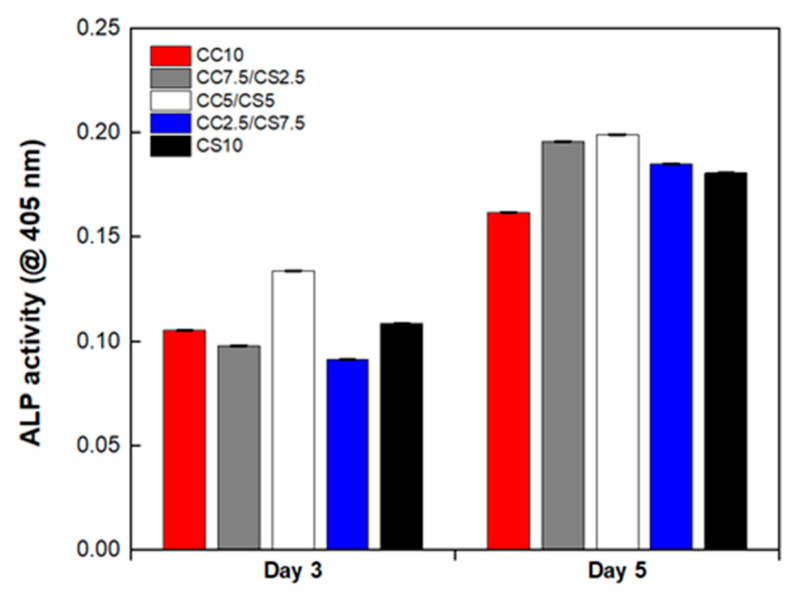
ALP activity of DPSCs seeded on the CSP-CPC scaffolds.

Alkaline phosphatase, which is recognized as an early phenotypic marker, serves as an important parameter for the evaluation of cell differentiation. The ALP activity was determined at 7 and 10 days in culture (Figure 10). The ALP activity of CSP-CPC scaffolds increased over time. Higher cell differentiation was induced by the presence of multiphasic Ca-P formation in the constructs. The observation of porosity with a uniform distribution in macroporous CPC scaffolds containing polyphasic Ca-P was found to facilitate the differentiation of osteogenically induced stem cells. The ratio composition of HA and β-TCP promotes stem cells by increasing ALP activity.

**Figure 11:**
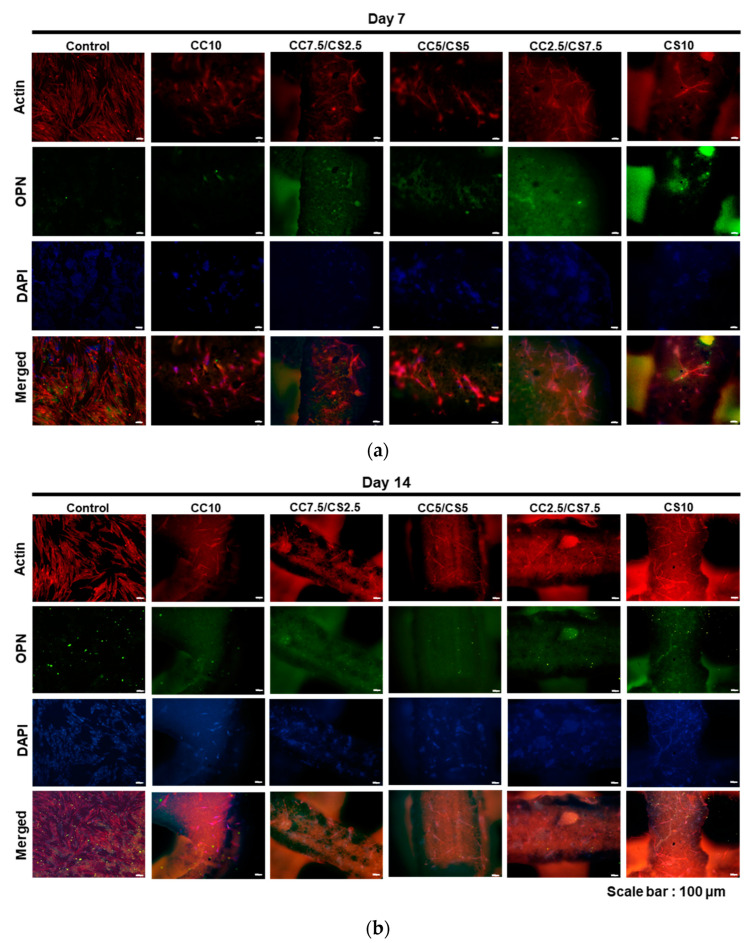
Immunofluorescence images of CSP-CPC scaffolds. Actin, OPN, and DAPI were exhibited as red, green, and blue. (**a**) 7 days. (**b**) 14 days.

To investigate the osteogenic differentiation of DPSCs into CSP-CPC scaffolds, immunocytochemistry (ICC) was performed to observe the osteopontin (OPN) expression, which may increase during the osteogenic differentiation of cells (Figure 11). DPSCs were cultured in osteogenic medium for 14 days (Figure 11b). Overall, OPN expression was elevated in all experimental groups of CSP-CPC scaffolds.

### 3.3. In Vivo Test Results

**Figure 12:**
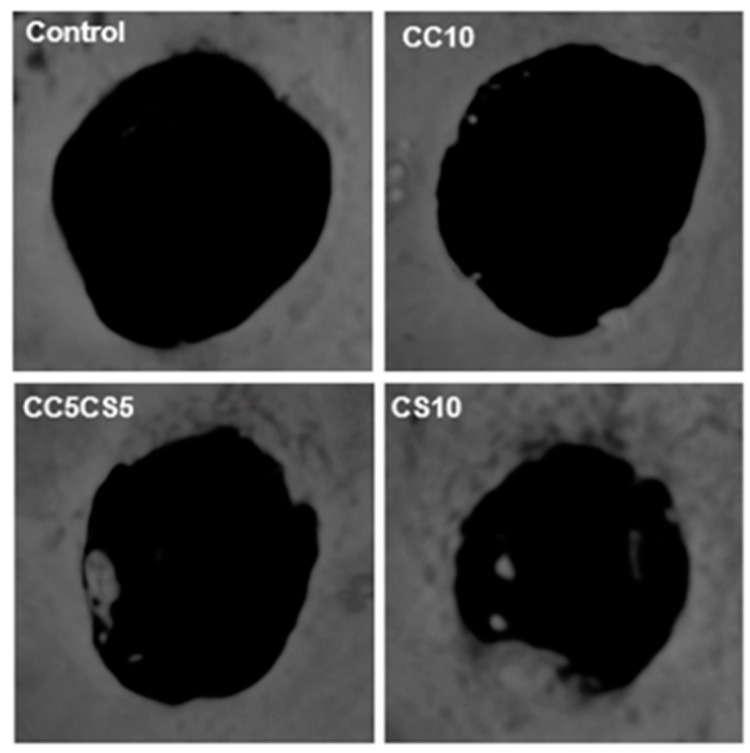
Micro-CT of a mouse calvarial defect model implanted with a 3D scaffold after 6 weeks of healing.

**Figure 13:**
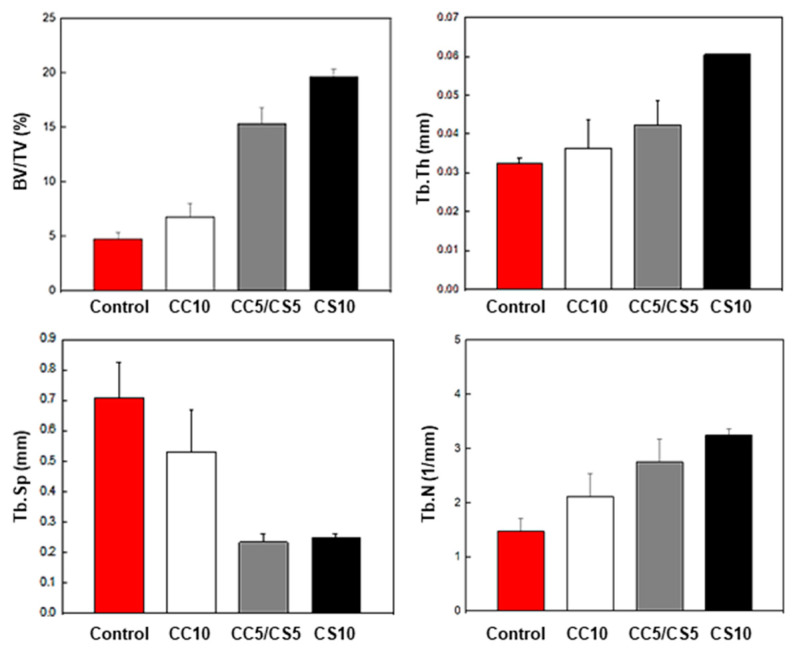
Quantitative measurement of the newly formed bone, and micro-CT images.

In the animal study, CSP-CPC scaffolds maintained their structural integrity throughout the surgical procedure and were securely attached to the defect. The CSP-CPC scaffolds also exhibited good wettability. Figure 12 shows the micro-CT scan of a mouse calvaria that was implanted with a 3D scaffold and allowed to heal for 6 weeks. Bone formation increased in proportion to the percentage of cockle shell used when measuring newly formed bone. This finding suggests that the cockle, which is naturally composed of calcium carbonate, creates a bone-like setting and exhibits greater bone mineralization than synthetic calcium carbonate (Figure 13).

## 4. Conclusions

In this study, we fabricated pastes of CSP-CPC scaffolds and verified them through infrared peak shift and degradation tests. The CSP-CPC scaffolds were successfully produced using the extrusion method with a narrow nozzle rather than existing CPC 3D printing. On CSP-CPC scaffolds, DPSC cells showed the highest viability, migration, proliferation, and differentiation in CC2.5CS7.5. In animal testing, the researchers noted greater bone formation around the CSP-CPC scaffolds due to the presence of a bone-like environment provided by the CSP-CPC scaffolds. Thus, the CSP-CPC scaffolds demonstrate significant potential as a substitute for bone tissue regeneration and can generate considerable value by utilizing discarded cockle shells, which are a byproduct of the fishing industry.

## Figures and Tables

**Table 1:** Concentrations of basic components.

	Basic Components	Concentration (*w*/*v* (%))
Powder	β-TCP	60
	Hydroxyapatite	4
	DCPA	26
	Classification	10
Solution	Sodium hydrogen phosphate	4
	PEG	15
	Hyaluronic acid	1
	Lactic acid	2
Classification	Calcium carbonate (%)	Cockle shell powder (%)
CC10	10	0
CC7.5CS2.5	7.5	2.5
CC5CS5	5	5
CC2.5CS7.5	2.5	7.5
CS10	0	10

**Table 2:** EDS tables of CSP-CPC scaffolds.

	CC10		CC7.5CS2.5		CC5CS5		CC2.5CS7.5		CC10	
Element	W_t_ (%)	Atomic (%)	W_t_ (%)	Atomic (%)	W_t_ (%)	Atomic (%)	W_t_ (%)	Atomic (%)	W_t_ (%)	Atomic (%)
C	23.73	33.16	23.39	32.37	26.97	37.72	34.27	47.47	28.43	39.63
N	0.00	0.00	3.65	4.33	3.26	3.91	0.00	0.00	1.52	1.82
O	49.18	51.58	47.27	49.11	39.53	41.50	32.80	34.11	39.71	41.56
Na	1.90	1.39	0.72	0.52	0.46	0.34	1.00	0.72	2.01	1.47
Al	2.90	1.81	3.37	2.07	4.71	2.93	6.84	4.22	2.62	1.63
P	22.28	12.07	21.61	11.59	25.07	13.60	25.10	13.48	25.71	13.90
Total (%)	100	100	100	100	100	100	100	100	100	100

## Data Availability

The data presented in this study are available on request from the corresponding author.
